# Male circumcision for HIV prevention: current evidence and implementation in sub-Saharan Africa

**DOI:** 10.1186/1758-2652-14-49

**Published:** 2011-10-20

**Authors:** Richard G Wamai, Brian J Morris, Stefan A Bailis, David Sokal, Jeffrey D Klausner, Ross Appleton, Nelson Sewankambo, David A Cooper, John Bongaarts, Guy de Bruyn, Alex D Wodak, Joya Banerjee

**Affiliations:** 1 Department of African-American Studies, Northeastern University, Boston, MA, USA; 2 School of Medical Sciences, University of Sydney, Australia; 3 Research & Education Association on Circumcision Health Effects, Bloomington, MN, USA; 4 Behavioral and Biomedical Research, Family Health International, Research Triangle Park, NC, USA; 5 Department of Medicine, University of California, San Francisco Department of Public Health, USA; 6 College of Professional Studies, Northeastern University, Boston, MA, USA; 7 Makerere University College of Health Sciences, Kampala, Uganda; 8 Kirby Institute, St Vincents Hospital and University of New South Wales Sydney, Australia; 9 Population Council, One Dag Hammarskjold Plaza, New York, NY, USA; 10 Perinatal HIV Research Unit, New Nurses Home, Chris Hani Baragwanath Hospital, Johannesburg, South Africa; 11 Alcohol & Drug Unit, St Vincent's Hospital, Sydney, Australia; 12 Global Youth Coalition on HIV/AIDS, Pretoria, South Africa

## Abstract

Heterosexual exposure accounts for most HIV transmission in sub-Saharan Africa, and this mode, as a proportion of new infections, is escalating globally. The scientific evidence accumulated over more than 20 years shows that among the strategies advocated during this period for HIV prevention, male circumcision is one of, if not, *the *most efficacious epidemiologically, as well as cost-wise. Despite this, and recommendation of the procedure by global policy makers, national implementation has been slow. Additionally, some are not convinced of the protective effect of male circumcision and there are also reports, unsupported by evidence, that non-sex-related drivers play a major role in HIV transmission in sub-Saharan Africa. Here, we provide a critical evaluation of the state of the current evidence for male circumcision in reducing HIV infection in light of established transmission drivers, provide an update on programmes now in place in this region, and explain why policies based on established scientific evidence should be prioritized. We conclude that the evidence supports the need to accelerate the implementation of medical male circumcision programmes for HIV prevention in generalized heterosexual epidemics, as well as in countering the growing heterosexual transmission in countries where HIV prevalence is presently low.

## Review

Implementation of male circumcision (MC) for HIV prevention in sub-Saharan Africa remains disappointingly slow despite its proven efficacy of greater than 60% based on the results of three randomized controlled trials (RCTs) conducted in the region [1-3]. These data received support from a Cochrane review [4] and confirm more than two decades of data from observational studies [5]. An as-treated meta-analysis for the 15 observational studies that adjusted for potential confounders gave a summary risk ratio indicating a protective effect of 65% that was identical to the initial findings from the three RCTs [6,7]. Another meta-analysis of the RCT data reported a relative risk reduction of 56% [8].

In a meta-analysis of 13 studies, 85% of which were from sub-Saharan Africa, a 58% protective effect was noted (53% for general populations and 69% for high-risk populations) [9]. In this report, protection was 57% for the RCTs and 61% for observational studies (cohort studies 71% and case control 46%). In addition, if MC status was ascertained by self-report, the protective effect was 45%, but if by direct genital examination in the clinic, it was 65%. These authors pointed out that the current data on MC satisfy six of the nine criteria of causality as outlined by Sir AB Hill, namely strength of association, consistency, temporality, coherence, biological plausibility and experiment [10].

With these definitive results, key international health bodies [11,12] and numerous governments of countries most affected [13,14] have formulated affirmative policies on MC for HIV prevention. There is now a consensus among most experts in the HIV/AIDS scientific community that MC, although not a "magic bullet", is a critical component in the "tool box" of HIV prevention approaches. Crucial to the effectiveness of MC policy is an understanding of how effective MC will be in HIV reduction, and as a corollary to this, the level of importance that heterosexual transmission plays in overall HIV transmission in a population.

Historically, transmission of HIV has been attributed to four main modes: sexual intercourse, transfusion, parenteral and perinatal acquisition [15]. In light of this, multiple types of intervention strategies (behavioural, structural and biomedical) have been advocated [16]. While scientists seek to provide the evidence base, public policy makers must evaluate logically where the preponderance of evidence lies, and make correct decisions based on a reasonable assessment of such evidence [17]. Urgent calls have been made by experts and advocates to accelerate HIV prevention scale up in line with the prevention principles [18,19]. However, in some instances skepticism about the evidence has led to hesitation, delays and inaction, leading to misery and, as in South Africa, needless death for hundreds of thousands from failure to expeditiously implement programmes that work [20].

In the sub-Saharan African setting, the established convention is that heterosexual transmission is the primary driver for the HIV epidemic. Recently, however, some have argued that current HIV prevention interventions are based on "insufficient information" on modes of transmission and what works [21]. We agree that there is a need to continually evaluate and update knowledge on HIV transmission and what works in prevention so as to better inform and reinforce policy making and implementation. Therefore, in reinforcing the policy imperative for MC as a proven method for prevention of heterosexual HIV transmission, we first review the state of knowledge on modes of HIV transmission in sub-Saharan Africa. We then assess the strength of current evidence for MC in protecting against HIV infection, before analyzing current MC implementation programmes in the region. Finally, we highlight some of the outstanding issues and call for an acceleration in MC implementation as an evidence-based strategy to stem the HIV/AIDS epidemic.

## What we know about the drivers of HIV infection in sub-Saharan Africa

According to the latest Joint United Nations Programme on HIV/AIDS (UNAIDS) epidemic update report, of the 33.3 million people living with HIV/AIDS worldwide at the end of 2009, 92.5% were adults [22]. About half were women and 67.6% live in sub-Saharan Africa, where women comprise about 60% of cases [22]. Of total infections globally, 2.5 million (approximately 7.5% of the total) were in children (aged younger than 15 years), of whom 92% live in sub-Saharan Africa where they comprise 10% of all cases [22]. Although some children younger than 15 are sexually active, the prevalence estimates show that 92.5% of cases globally and 90% in sub-Saharan Africa are in the adult population [22].

While this in itself does not necessarily show association with sexual activity, the preponderance of infection in adults can only be explained by adult-specific factors not affecting children. HIV distribution by sex and across age groups are clearly consistent with sexual behaviour as the main mode of transmission, as shown by the significant and sudden increase in HIV in those older than 15 years [22,23].

Consistent evidence for a major role of sexual intercourse in transmission of HIV has been provided by numerous modelling studies [24-28] and ecological observations, published by the World Health Organization (WHO) and UNAIDS in their annual report [22], as well as in national estimates [29]. These data are collected according to global norms [30,31] whose methods are continually refined [32-35].

The effect of sexual transmission likely lie in context-specific factors confounding host and agent alike. These include stage of the disease, associated viral load, other biological factors [24,36-40], as well as the socio-economic and policy context [20,41]. Credible research shows that the key initial drivers of the sexual transmission were a synergistic relationship between promiscuous practices, coupled with individual-level biological factors, namely sexually transmitted infections (STIs) (in particular, genital ulcer disease, syphilis and HSV-2) and lack of MC [40-44]. These factors also help explain the differences in rate of spread across the continent [42,45].

Among these, the role of multiple and concurrent partnerships (unstructured casual sex and polygyny) is well documented in many sub-Saharan Africa countries [46-51]. This is demonstrated, for example, in one of the most detailed studies, involving 179 focus groups and 116 in-depth interviews with diverse groups of people (male/female, young/old, urban/rural) in typical day-to-day settings in 10 countries of southern Africa that have high generalized HIV epidemics [52]. Modelling and network theory reveal dynamics of exposure [53,54]. Nevertheless, there is conflicting evidence on the extent to which multiple and concurrent partnerships drives the epidemic, as shown in recent assessments of the existing literature by Lurie and Rosenthal [55] and Sawers and Stillwaggon [56], as well as a debate hosted on 27 October 2010 by the United States Agency for International Development (USAID) and the World Bank among opposing sides [57].

While heterosexual behaviour plays a leading role in HIV transmission in sub-Saharan Africa, it is fair to ask what proportion of infections is from non-sexual drivers. These include: unsafe injections in medical and non-medical settings [58,59], injecting drug use and blood transfusion [60-62], mother to child transmission [22,61,63,64], men who have sex with men (MSM) [65,66] and cultural practices [67,68], such as unsafe traditional MC and female genital cutting [69-72]. While some argue that these play a significant role in sub-Saharan Africa [21,68,73,74], such evidence is largely circumstantial [62,75] and the level of such exposures are altogether too low to fuel a generalized epidemic [75-77]. One crucial illustration of this comes from a modelling approach in use since 2003 [61], namely the Modes of Transmission (MoT) approach. Developed by the UNAIDS Reference Group to help country-level policy makers respond to the epidemic and prioritize interventions, the MoT approach provides a robust means for estimating patterns of adult HIV transmission through different routes [61].

To date, MoT analyses have been conducted in Kenya [61], Lesotho [78], Swaziland [79], Uganda [80] and Zambia [81]. They show that sexual behaviour accounts for 94.1%, 97.0%, 94.0%, 99.6% and 99.8% of new infections, in each respective country, with the categories of multiple partnerships and partners of multiple partnerships contributing more than half of all cases in Lesotho and Zambia (Table 1). On the other hand, the population of MSM in sub-Saharan Africa is not known, partly due to laws prohibiting this behaviour in most countries, although HIV prevalence in MSM is, however, high [65]. A systematic review estimated that the MSM route makes an approximately 15.7% contribution to HIV prevalence in sub-Saharan Africa [82]. In one MSM cohort, in Mombasa, Kenya, HIV prevalence was 43% in men reporting exclusive MSM relations compared with 12% in men reporting sex with both men and women [83].

**Table 1:** Incident HIV infections by modes of transmission in five sub-Saharan Africa countries

	% share of modes of HIV transmission in five countries				
	
	Uganda (2008)	Kenya (2006)	Zambia (2008)	Swaziland (2008)	Lesotho (2008)
Injecting drug users (IDUs)	0.28	4.84	0	1.1	0
Partners of IDU	0.01	0.2	0	0.1	0
**Sex workers (SW)**	**0.91**	**1.25**	**0.75**	**3**	**0.47**
**SW clients**	**7.83**	**10.48**	**4.04**	**4.7**	**0.59**
**Partners of SW clients**	**1.81**	**1.1**	**1.81**	**2.6**	**1.68**
**Men who have sex with men (MSM)**	**0.61**	**4.49**	**0.99**	**3.6**	**2.89**
**Female partners of MSM**	**0.1**	**0.64**	**0.05**	**0.5**	**0.5**
**Multiple partnerships (MP)**	**23.73**	**18.31**	**33.96**	**13.4**	**31.04**
**Partners' MP (PMP)**	**21.76**	**27.74**	**37.03**	**20.8**	**27.45**
**Mutually monogamous heterosexual sex**	**42.89**	**30.14**	**21.19**	**49.8**	**35.15**
Medical injections	0.06	0.55	0.17	0.01	0.04
Blood transfusions	0	0.24	0.02	0.02	0

Bold text indicates sexual transmission [61,78-81].

In contrast, in Kenyan, Ugandan and Zambian studies, infections arising from blood transfusion were insignificant (Table 1). Furthermore, a review of Demographic and Health Surveys data from 10 countries indicated that although having had a blood transfusion increases risk of HIV infection among women (but not men) in Cameroon and Uganda, blood transfusions are rare [62]. While non-sexual routes are relatively minor, they merit continued monitoring [21,74,84]. Nonetheless, at present, evidence-based means of reducing heterosexual transmission should be prioritized [85]. One of these is male circumcision.

## Efficacy of male circumcision for HIV prevention: an update on the evidence

As one of the oldest surgical procedures known to humankind and the most widely practiced surgery worldwide, male circumcision has served religious, socio-cultural and health purposes [69,86-88]. Since the suggestion in the 1980s that MC might prevent HIV infection [89-91], numerous ecological, case-control and cohort studies, reviews, systematic reviews and meta-analyses have established that MC significantly reduces the risk of heterosexual HIV infection [6-9,69,92-94]. The meta-analysis by Weiss *et al *of 27 observational studies to the late 1990s showed a reduced risk in 21 studies [7]. In 15 studies that adjusted for confounding factors, adjusted relative risk reduction was 0.42 (95% CI 0.34-0.54) [7]. A Cochrane systematic review in 2005 assessing the quality of 37 studies of MC and HIV noted that while the different methodologies showed varying results, the protective effect of MC was supported consistently [93].

In 2005 and 2007, the efficacy of MC in HIV prevention was verified beyond reasonable doubt by results from three large RCTs, the gold standard of epidemiological research, these being conducted in South Africa, Kenya and Uganda [1-3]. Acceptance by the international health community quickly followed [11-14]. In 2009, the Cochrane committee concluded that MC for HIV prevention was supported and no further trials were required [4]. More recently, a systematic review of 37 late-phase RCTs of various HIV prevention interventions found MC to have a stronger efficacy in preventing HIV infection than vaccines, microbicides and treatment of STIs [95]. An important development in MC documentation has been the initiation by UNAIDS, WHO and others of an online resource centre [96].

Research on acceptability of MC at the population level in sub-Saharan Africa countries in 14 studies in nine countries showed varied results, but was generally high among men and women [97,98]. Continued assessment of impacts, challenges and opportunities [99,100] support the urgency of accelerating the implementation of MC for HIV prevention [101,102]. In 2010, the US-based Center for Global Health Policy called for "aggressive scale up" of MC based on the evidence for its ability to prevent HIV infection [103]. The quality of the evidence supporting MC is "conclusive" [7], making MC a sound recommendation for public health [104]. Such evidence calls for skepticism to be dismissed [17].

## Biological mechanism of male circumcision in mediating HIV infection

Several suggestions have been made to explain the vulnerability of the foreskin to HIV infection. The inner epithelium of the foreskin is mucosal, has been found to lack protective keratin and to contain Langerhans cells and T cells that express the HIV receptor, CD4 [87,105-109]. In an early study, HIV was taken up readily by the inner, but not the outer foreskin epithelium in explant culture [106]. Nearly a decade later, Ganor and colleagues developed two new excellent models of the foreskin epithelium: an improved explant model and a 3D immuno-competent *in vitro *model [108]. Their human adult *ex vivo *foreskin explant model showed that Langerhans cells and dermal T cells in the less-keratinized inner foreskin have a significantly higher density than in the outer foreskin. When the foreskin was exposed to mononuclear cells highly infected with HIV, but not free HIV, virions were found in the epidermis of the inner foreskin within one hour, demonstrating that Langerhans cells can efficiently transfer HIV to T cells [108].

In another review, Ganor and Bomsel suggested that the main pathway for HIV entry was driven by molecular signals, such as chemokines [109]. Findings of no difference [110] in, or greater [111], keratin thickness of the outer versus the inner foreskin or in susceptibility to HIV [112] have been dismissed as products of postmortem changes and technical artifacts [109]. Foreskin aspects relevant to HIV infection include the skin surface area, the microbiologic environment, HIV-1-susceptible cells and tissue structure, although more research is needed to determine the relative contribution of each [113].

Besides the ease of infection by inner epithelial cells to HIV, HIV is suspected of infecting the body via tears in the fragile inner surface of the foreskin and frenulum, which are also susceptible to infection by other STIs [43,87,105]. STIs hamper the ability of langerin in Langerhans cells to protect against HIV [107]. Thus the vulnerability of the foreskin to HIV infection lends biological support to the extensive epidemiological evidence for the protection MC confers against HIV infection in men during heterosexual intercourse.

## The relationship between MC and HIV: evidence and issues from population-based surveys

The highly acclaimed RCTs [1-3] would not have taken place had there not been extensive observational evidence in place already attesting to the ability of MC to prevent HIV transmission. Nevertheless, RCTs have been overvalued in medical studies and, by themselves, they yield insufficient evidence for policy and must be supplemented by observational evidence [5]. Population-based surveys, in particular, the Demographic and Health Survey (DHS) and the AIDS Indicator Survey, have been cited frequently because of the insights they provide into the patterning of HIV and MC in sub-Saharan Africa [49,51,94,114,115].

Some have, however, disputed the association of HIV prevalence and MC levels in such surveys. For example, Gisselquist *et al *refer to DHS data showing higher prevalence of HIV in circumcised men in seven of 13 sub-Saharan Africa countries [21]. In contrast, the ecological analysis of 118 developing countries by Drain and colleagues showed that high MC prevalence was strongly correlated with low HIV prevalence, independent of religion [94].

Furthermore, a recent cross-sectional analysis of DHS data for 18 countries across sub-Saharan Africa from 2003 to 2008 involving 70,554 males aged 15 to 59 years confirmed that being uncircumcised was significantly associated with risk of HIV infection (OR 4.12; 95% CI: 3.85-4.42) and that risk increases with number of lifetime partners [115]. In light of the probable conflict in interpretation, Wamai *et al *[116] have warned that DHS data must be used with caution because of their widely acknowledged inherent methodological problems, which Gersovitz asserts need to be overcome in order to improve reliability [117].

DHS data are, moreover, often bidirectional, indicating contrasting and context-specific effects. In Tanzania, for example, circumcision in men is higher in the upper quintiles of education and wealth, and such men have more sex partners [118]. So, not surprisingly, HIV prevalence in Tanzania, and numerous other countries across sub-Saharan Africa, is higher in people with higher education and income [119,120]. Furthermore, this relationship is not necessarily linear and can change over time [41]. The higher risky behaviour of such men, such as being more likely to have concurrent partners than uncircumcised men, would explain why, in certain settings, they have a high HIV prevalence despite being circumcised. This was pointed out in a recent analysis of surveys in 21 countries in sub-Saharan Africa [49].

Since DHS data involve self-reported surveys, multiple factors have to be considered when examining the relationship between MC and HIV prevalence. These include risky sexual behaviour, time of MC, whether circumcision was complete, partial or performed at all, marital status, education, wealth and patterns of residence (urban vs. rural). As an example, a study in Uganda by Gray *et al *of a large cohort of HIV-negative men found that MC significantly reduced HIV acquisition (unadjusted RR = 0.61; 95% CI = 0.37-0.97), but the protective effect was lower for post-pubertal circumcision (i.e., after 12 years of age); in Muslims, it was further confounded by cultural and behavioural factors [92]. In another example, a cross-sectional study in Kenya, Lesotho and Tanzania found that while the protective effect of MC in *adolescents *was only "probable", in *adults*, the association of MC with lower HIV infection was unequivocal, indicating a protective effect in males who were more likely to be sexually active [121].

There is a further point to note from ecological observation. Molecular clock analyses indicate that HIV has existed for about 70 years and may have originated in or near Cameroon (HIV-1), Guinea-Bissau (HIV-2) and the Congo [122,123]. Yet none of these countries, where most men are circumcised [69], have had adult prevalence rates as high as those observed in eastern and southern Africa [22,39]. Hence, it could be reasonably concluded that structural features of wealth and poverty patterning behaviour [41], reported practice of multiple and concurrent partnerships [49,52], couple discordancy [124], prevalence of other STIs [40,42,44] and geographic variations in MC [69,94] have synergized to provide the "perfect storm" for the HIV epidemic in sub-Saharan African countries with low MC prevalence [43,91].

## Male circumcision for HIV prevention: saving lives and costs - the policy imperative

Unlike other HIV prevention strategies, MC is a one-time procedure conferring potentially lifelong protective benefits, so making it a highly cost-effective, life-saving intervention, as revealed by several studies subsequent to the RCTs [99,125-130]. For example, a study by the UNAIDS/WHO/SACEMA Expert Group on Modeling the Impact and Cost of Male Circumcision for HIV Prevention found that one HIV infection would be averted for every 15 circumcisions at a cost of US$150 to US$900 over a 10-year time horizon [130]. The population-level impact of MC in reducing HIV incidence at significant cost-savings is potentially enormous, as shown in an early modelling study [104].

The Male Circumcision: Decision Makers' Program Planning Tool (DMPPT), developed recently by USAID's Health Policy Initiative in collaboration with UNAIDS, has estimated the cost and impact of scaling up MC services [131]. Using this model, an analysis of 14 priority countries in eastern and southern Africa found that scaling up MC services to cover 80% of all adult men and newborn boys would, over the period 2009-2015, avert more than 4 million new adult HIV infections at a cost of US$2.5 billion [132]. This would yield total net saving on cost of antiretroviral therapy (ART) of US$20.2 billion over the same period [132].

In the DMPPT model, annual costs for implementation were projected to increase in the early scale-up phase due to increased demand, peaking in 2012 and declining thereafter, to level off at around $100 million by 2015. Even countries with moderate HIV prevalence, such as Rwanda, could reap significant savings in costs relative to lifetime HIV treatment [133]. Furthermore, the cost-effectiveness of MC, even in non- or low-generalized HIV settings, increases when the procedure is performed in newborns [134,135].

Despite being targeted at sexually active men, MC provides important direct and indirect benefits to women and children. For example, it was estimated that in high-prevalence areas in Kenya and Zimbabwe, "circumcision confers a 46% reduction in the rate of male-to-female HIV transmission", with the effect of the intervention "doubling the number of infections averted among women" [136]. On the other hand, a RCT in Uganda of sero-discordant couples in which the man was HIV-positive was discontinued for futility after 21.7% of women in the intervention group and 13.4% in the control group became infected [137]. This difference was not, however, statistically significant, and many men disobeyed instructions by resuming sexual intercourse before healing was complete [137]. More recent findings from a prospective multinational study in a similar sero-discordant population showed "no increased risk and potentially decreased risk" of infection due to MC to the female partners [138].

Since women in sub-Saharan Africa show high acceptability of MC as part of comprehensive strategies for HIV prevention, they can play an important role in the adoption and implementation of MC by changing male norms and in promoting infant MC [97,98,139]. By lowering infection in men and thence women, MC will reduce overall infection rate and lower the number of children being infected by their mother. Infant MC is, moreover, simpler, more convenient, entails lower risk and provides considerable savings in cost when compared with circumcision at a later age, including the cost of treatment over the lifetime for HIV-infected people [132-135,140,141]. As an example, one study in the USA indicated a 16% reduction in lifetime risk of HIV infection in all males when circumcision is done in infancy [134].

The cost savings from circumcision of boys early in life is considerably greater than this because they enter the sexually active period of their life with a reduced risk of various STIs [87,142-146]. In the Ugandan RCT of MC and HIV, MC was associated with a 25% reduction in prevalence in herpes simplex virus type 2 (HSV-2), 35% lower human papillomavirus (HPV) [147] and significantly reduced ulceration, trichomonas and bacterial vaginosis [148]. In the South African MC trial, low-risk HPV prevalence was 8.5% in the intervention arm compared with 15.8% in the control arm [149]. The strong protective mechanism by which MC prevents STIs in men likely involves both cellular and anatomical factors [105,147].

That MC affords protection against HIV and multiple STIs in heterosexual men and their female sexual partners, and thereby their children, is not in doubt. On the other hand, the effect of MC in preventing HIV in MSM is less certain. In a South African study, HIV in MSM was 80% lower if they were circumcised [150]. A meta-analysis of studies from countries worldwide showed 29% protection only for MSM who adopt primarily the insertive role [151]. This was 73% in a Cochrane analysis [152]. Not included was a recent study of MSM in the high-prevalence setting of Andhra Pradesh, India, where 18.6% of MSM were HIV positive [153]. Although HIV was 70% lower in circumcised receptive-only MSM, this was probably a result of homophily. Further research in sub-Saharan Africa that takes into account social and sexual networks in MSM is needed [152,153].

With the current strong evidence that MC protects against HIV and several common STIs, questions that are important for policy consideration have arisen. These include adverse effects, acceptability, risk compensation, reduced efficacy due to early return to sex after MC, disinhibition, long-term consequences and external validity, as well as ethical issues. These have been addressed in numerous publications [11,12,87,97,98,116,154-156], none of which regard these considerations as representing a basis for rejecting MC as part of HIV prevention strategies. For example, the arguments of external validity raised by Green *et al *[157] ignore long-standing evidence from observational studies [5] and have been strongly refuted as unfounded [158]. In other examples, studies on disinhibition [159] and risk compensation [155,160] showed no increase in risky sexual behaviour [160] or early resumption of sex [155].

Follow-up data of the Kenya RCT [2] indicated an ongoing increase in the protective effect of MC against HIV infections at 42 months [161] and 54 months [162]. By five years, the protective effect reached 73% in the Ugandan trial [163]. These results suggest that the positive effect of MC will continue [158]. However, implementation of national MC programmes triggered by the RCT findings did not begin until 2008 [7], starting in Kenya [164], and thus the long-term population impact remains to be observed in those particular areas. In light of that, it is imperative to continue monitoring sexual behaviour after circumcision for continued assessment of long-term positive impact.

## Current state of practice in MC interventions in sub-Saharan Africa

Following the recommendation by global health agencies that MC be adopted as one of the critical tools for HIV prevention in high-prevalence generalized heterosexual epidemics [11,12], WHO and UNAIDS developed operational guidelines for scaling up MC services [165]. Programmatic development has, however, been slow, in large part as a consequence of suboptimal funding.

In 2008, researchers argued that the international community was not committing enough resources to MC commensurate with the available evidence on what works [166]. These authors noted that the 5% allocated for MC, from an overall budget of $3.2 billion that UNAIDS had estimated was needed to achieve universal coverage for HIV programmes by 2010, fell far short of the estimated need and demand for MC, especially given its demonstrated efficacy relative to other interventions. Table 2 summarizes the current state of MC intervention policy strategies, projected cost savings and infections averted, as well as MC provision to date in the 14 priority African countries. It can be seen that programmatic development of MC to date is ongoing in all countries, but differs markedly in extent [13,101,167].

**Table 2:** Design and implementation of MC services for HIV prevention in 14 priority countries in east and southern Africa, 2011

*Country*	*HIV prevalence (%)*	*Men circum-cised (%)*	*Policy framework*	*Implementation strategy, plan status*	*MC delivery structure*	Potential infections averted by scaling up MC to 80% by 2015 and maintain rate through 2025*	*Total Net Savings, 2009-2025 (US$)*	*Circum-cisions to date*	*Estimated number of MCs needed to reach 80% target*	*Achievement towards 80% target (%)*
Botswana	17.6	11.2	MC as part of existing HIV prevention policy	In place	Services integrated in existing HIV prevention strategies	62,773	248 million	11,197	345,244	3.2
				*Phased scale-up goal, 80% of 0-49 years HIV-negative men by 2014*						
Ethiopia	1.4 - National	93 - National	MC as an additional HIV prevention strategy. Regional MC Task Force is to be established; draft regional MC strategic direction document under finalization.	Under development	MC to be provided in 100% of medical facilities in Gambella (one hospital and 25 health centres)	1,479	5.8 million	5,786	100,000	5.8
	6.0 - Gambella	46 - Gambella		*Target to provide services in 100% of healthcare facilities in Gambella Region*						
Kenya	7 - National	86- National	MC policy in place: 'National Guidance' for MC	In place	Stand alone and integrated, mobile clinics; prison services	73,420	247 million	232,287	860,000	27
	15.4 - Nyanza	48 - Nyanza		*Target to reach 80% of 15-49 year old men (1.1 million men) and newborns by 2013*						
Lesotho	24	52	MC policy in place	In place	MC to be integrated in HIV prevention services focused in MNCH settings	106,427	618 million	4,000	376,795	1
				*Launched in 2010*						
Malawi	11	21	In place	National operational plan includes voluntary MC	Currently offered by free-standing clinics. Scale-up structure not yet developed	240,685	1.2 billion	3,119	2,101,566	0.1
Mozambique	12	52	Formal policy developed	MC included in operational plan for HIV prevention	MC services available on demand; adolescent and neonatal MC are planned.	215,861	1.5 billion	7,733	1,059,104	0.7
				*Rollout in pilot sites*						
Namibia	13	21	MC policy approved	In place	Stand-alone, mobile services are being considered. Plans to integrate into hospital services.	18,373	120 million	1,987	330,218	0.6
				*Rollout in pilot sites*						
Rwanda	3	12	Formal policy in development. Detailed operational plan in place	In place	Formal scale up started in the military. Plans to integrate into standard HIV prevention services.	56,840	200 million	1,694	1,746,052	0.1
				Detailed operational plan being rolled out						
										
South Africa	18	42	Draft policy in place, under finalization	In place	Facility based, and stand-alone centres and camps, scale up from Orange Farm to 143 sites	1,083,869	6.5 billion	131,117	4,333,134	3.4
				*Currently being scaled up nationwide*						
Swaziland	26	8.2	Policy adopted by cabinet	In place	Formal scale-up of integrated services started; dedicated "circumcision Saturdays'	56,810	332 million	18,869	183,450	13.3
Tanzania	5.7	67	Policy under way	Under development. Plans to target 8 regions with high HIV and low MC prevalence	Scale-up demonstration sites, MOVE strategy recommended in the public sector	202,900	966 million	18,026	1,373,271	1.4
Uganda	6.4	25	Policy in place	In place	Piloted in the military and a mobile site, plans to integrate into routine services	339,524	2 billion	9,052	4,145,184	0.2
Zambia	14	12.8	Cabinet approved MC as part of HIV prevention policy	In place	Multi-sectored approach focused on military, police, prisons, and neonatal services	339,632	2.4 billion	81,849	1,949,292	4.2
				*Target of 250,000 MCs a year; MC sites to increase to 300 by 2014*						
Zimbabwe	14	10	Policy in place	Under development (2010-2014)	Services offered through mobile and free-standing sites and in public health clinics. Nationwide neonatal MC planned	565,751	3.8 billion	13,977	1,912,595	0.7

*Notes and data sources: *Ethiopia MC data (personal communication, Hannah Gibson, Country Director Jhpiego, Ethiopia) and estimated target [173]; Lesotho (4000 annual circumcisions before programme intervention) [169]; for Zimbabwe 30,000 circumcisions have previously been reported [170]; all other data [13,132,167,171].

* The 80% target in all three columns is for uncircumcised males 14-49 years.

Implementation in Kenya, the first country to commence, was spearheaded by a national task force on MC in 2008 [14]. Other countries have, or are in the process of developing similar policies, implementation guidelines and strategies. Some, like Kenya and Lesotho, have developed formal MC policies, while others, such as Botswana and Rwanda, have incorporated MC into existing HIV prevention policies. Translating science into policy is often challenging [168], and we acknowledge that development of documents and programmes through consultative and collaborative processes involving stakeholders in the health ministries, HIV/AIDS agencies, non-governmental organizations, academia and donor partners, as was the case in Kenya, can be time consuming.

It is nevertheless of concern that the numbers circumcised across the various countries three years after policy recommendations are very low relative to targets (Table 2). The latest WHO/UNAIDS report indicates cumulative circumcision figures up to 2010 since scale-up started in 2008 at 555,202, i.e., 2.7% of the 20.8 million target [167]. That 74% (410,904) of these occurred in 2010 alone indicates that the momentum is rising, but needs to accelerate still. As the DMPPT modelling indicates, to achieve the projected outcomes, the 14 countries will need to reach 12 million circumcisions at peak period in 2012 [132]. Accordingly, five countries (Malawi, South Africa, Tanzania, Uganda and Zimbabwe) would require at least one million circumcisions each in 2012 [132].

In most of these countries, MC prevalence varies by region and it is logical that, in the scale-up phase, programmes for MC deliberately target low MC localities, such as is occurring in Ethiopia, Kenya and Namibia. However, many of the current programmes are confined to small or pilot settings. Data available for Lesotho are pre-scale up [169]; for Zimbabwe, they are from several clinical sites [167,170]; and for South Africa, they have scaled-up from Orange Farm [171], where the RCT in that country was conducted, to over 140 sites [167]. In Gambella, Ethiopia, services are currently provided in one hospital and seven health centres (personal communication, Hannah Gibson, Country Director Jhpiego, Ethiopia).

With a growing demand for MC services and the potential cost and life savings, it is imperative that scale up be rapidly accelerated [103]. At the current rate of service provision, 12 million MCs by 2012 across the 14 countries are highly unlikely to be met, so putting in jeopardy many lives and failing to achieve the desired cost savings.

In Kenya, just 232,200 MCs have been completed [167], the largest number of any country. A speeded-up rapid-results initiative intervention during a 30-day period in 2009 conducted by 95 teams, each of four persons, at a range of 9.6-22.8 circumcisions per team per day, achieved 36,000 circumcisions (Robert Bailey, personal communication). A similar intervention conducted over five weeks during November-December 2010 achieved 51,000 circumcisions (Robert Bailey, personal communication). At these rates, Kenya would need several similar rapid-results initiatives to reach the national goal of one million circumcisions by 2013 [172]. Nevertheless, Kenya's programme is a model for other African countries and, if adopted, could advance the 2012 goal.

Many challenges stand in the way of implementing MC programmes. These include cost, need for training of health personnel, other health system barriers, the politics surrounding policy development, funding and changing socio-cultural perceptions and beliefs about MC [13,94,101,164,166,172,173]. In Gambella, Ethiopia, the regional hospital reportedly cannot meet even a small demand of 10 circumcisions per week due to staff shortages and lack of training [173].

Currently, the most informative assessment of MC programmes comes from Kenya [164]. This report reveals that of 81 government health facilities surveyed in Nyanza (the target location of MC services), none had the capacity to implement the full package of voluntary circumcision outlined in the national guidelines [14]. Challenges included lack of a theatre, MC kits and supplies, medical personnel to perform the procedure, and data monitoring tools. Due to this, most of the reported 230,000 circumcisions were done by partner organizations largely in high-demand settings using mobile teams [164]. The Kenya programme offers many lessons for other countries.

Health provider training and service models being developed will need to be tailored around specific existing health systems and services infrastructure, HIV epidemiological profiles and determinants, as well as MC prevalence and demand. Reaching the estimated 100,000 men that need to be circumcised in Gambella, Ethiopia, for example, will require a massive increase in trained personnel to conduct the surgical procedure [173]. Since MC programmes are targeting healthy men, high standards for surgical staff training and post-operative care are essential. This includes strictly following established national and international guidelines for sterile surgical practice [11,12,14,144,165].

To increase the number of health personnel who can perform safe circumcisions, novel service models should be adopted. The rapid-results initiative pursued in Kenya is based on intensive mobilization of resources (human, equipment and financial) in high-demand settings through community approaches [164]. Models for Optimizing the Volume and Efficiency of MC Services ("MOVE") is an additional approach for meeting demand. Currently practiced in South Africa, it is focused on increasing the efficiency of staff and time by considering alternate surgical methods and modifying facilities for efficient use [174]. Consideration should also be given to promoting task shifting for nurses and clinical officers as per WHO guidelines [175]. Already in practice in Kenya [164] and Zambia [167], it is a component of proposals in several other countries, such as Namibia, Lesotho [13].

In some of the scale-up countries, traditional circumcisers, already used widely [72], can play a role in meeting demand [176], but only if they receive adequate certification for acceptable standards of surgical MC. On the other hand, as exemplified by the high (90%) preference among men and women for medical MC in a traditionally circumcising community from northern Tanzania, more efforts should be made to provide this medical service in a culturally appropriate fashion, so encouraging uptake [177].

Preliminary data are also becoming available on devices that could facilitate quicker and safer adult circumcision [178]. These include the Shang Ring [179] (which produced good results for safety and acceptability in a field test in Kenya [180]), circumcision template [181], the recently acclaimed PrePex system [182], and the Tara KLamp [183], for which further assessment is needed [178] after adverse effects were initially reported [184]. In an important development, WHO has provided a framework for clinical evaluation of devices for adult MC [185], in addition to those already recommended for infant MC [144].

## Where do we go from here?

While welcoming continued debate about what drives HIV in high-prevalence populations and what works in HIV prevention programmes, we echo the call made by experts and advocates four years ago [186] and more recently [103], including a political declaration of the United Nations [187], urging an acceleration in implementation of proven approaches, such as MC. There are multiple reasons for reiterating this call. First, while the incidence of HIV is now declining in many countries in sub-Saharan Africa, nearly 70% of new HIV infections globally remain in this region [22]. The rate of new infections therefore needs to decelerate much faster there if the crisis is to be stemmed. Towards that end, policy makers, researchers and practitioners should direct energy towards viable, practical and efficacious solutions in an accelerated campaign.

Second, MC could stem epidemics of HIV elsewhere than Africa. Based on current UNAIDS data, the main mode of infection globally (heterosexual transmission) is growing, as reflected in the increasing proportion of new HIV infections reported in women, for example, to 35% in 2009 from 21% in 1990 in Asia [22]. Although in the USA, UK, Russia, Canada, Australia and the Asian region, major exposure categories are MSM and injecting drug users, higher incident HIV trends in women and heterosexual contacts [22,188] should ring alarm bells [143,156,189]. Such recent trends are likely to be exacerbated by uneven and declining MC levels, especially in such countries as the USA and Australia, which traditionally, until the mid-1970s and early 1980s, had MC rates of more than 90% [190,191]. In Australia, it is heartening that infant MC is again rising [156].

In such settings as the USA, MC services are particularly crucial, especially in African-Americans [189], who comprise a disproportionately high number of persons living with HIV [192,193] and in whom perinatal infection per 100,000 infants is 12.3 compared with just 0.5 in white infants [194]. Furthermore, African-Americans have the highest heterosexual HIV rates [195], but national data show that they also have rates of MC lower than whites [190]. Therefore, given the current epidemiological trends, interventions need to focus on established patterns of transmission for which the population-level impact in reducing HIV infections will be high now and in the future.

Third, current evidence from RCTs shows that in comparison to a protective effect of 46% for prophylaxis [196], 39% for microbicides [197] and 31.2% for a vaccine [198], at the moment, MC, with a 60% or higher efficacy [4], is the most effective biomedical HIV prevention strategy in heterosexual men. Furthermore, MC will help reduce HIV in women [136] and children [140,141,143], as well as help lower risks for STIs [146-148] that exacerbate HIV risk [199,200].

As part of the internationally recognized priority interventions for stemming HIV [201], and given the current state of implementation, massive catch-up strategies for adult MC seem to be the better investment in the short term. Importantly, mainstreaming of neonatal MC as part of a long-term strategy is both logical and clearly more cost effective [133,134,140,143,156], and will help systematize MC practice and services provision in the primary healthcare system for future generations. Furthermore, the much-needed scale up in sub-Saharan Africa will require significant additional funds, reorientation of expenditure allocation, and better, more rational use of the already existing largesse [132,164,166].

Last, continued research that addresses other issues concerning MC will be valuable, in addition to those already underway in various countries [13]. In particular, careful research is needed to:

1. Regularly update the impact of MC on the HIV epidemic in the targeted areas by monitoring behavioural changes following MC.

2. Compare different surgical approaches, including the use of different low-risk devices for adult MC to further improve on this procedure, and the cost effectiveness of service models, such as the rapid-results initiative, task shifting and "MOVE" for accelerating delivery.

3. Explore novel hypotheses relevant to prevention messaging, for example, does MC make condom use easier and/or more pleasurable?

4. Evaluate how to best integrate MC messages into existing communications and prevention programmes.

5. Develop strategies to improve the safety of traditional MC practices and norms so that these can be incorporated into regular scale-up programmes without increasing overall risk in order to speed up MC programmes.

6. Examine the effect of MC scale up on the health services and health system resources (human and infrastructural), as well as integration of the practice in the formal healthcare system.

7. Establish the definitive biologic mechanism by which MC protects against HIV infection through the penis.

8. Assess the role of MC as a potential platform for promoting men's health, including participation by women in order to encourage couple sexual and reproductive health.

9. Evaluate the integration of routine newborn MC in maternal-child health programmes.

## Conclusions

Public health campaigns aimed at stemming the spread of HIV/AIDS should address all known transmission routes as specific epidemiological, resources and contextual factors demand. We support the continued promotion of the use of all effective methods. The effect of doing so will be cumulative. We realize that MC definitively disrupts the major mode of HIV transmission in sub-Saharan Africa. We also realize that historical, cultural and political controversies surrounding MC [86,88] may provoke passionate debates. However, as Collins argues, it is imperative that values underpinning scientific thought form the centre of public policy interventions [17].

Given the present body of evidence, and contingent on certain pre-conditions (e.g., that MC is conducted by a qualified practitioner, under acceptable conditions of hygiene, in the absence of contra-indications), at this point in time, it is clear that medical MC in infancy, childhood or adulthood produces far greater good than harm. We urge policy makers to more urgently facilitate implementation of MC as a public health measure to stem the growing heterosexual transmission of HIV worldwide and, in sub-Saharan Africa, to more quickly reduce future epidemics. Not only is MC highly efficacious against HIV acquisition, but it also confers multiple other health benefits, thus making it quite rightly a "surgical vaccine" for the 21^st ^century [1,87,103,116,154].

## Competing interests

The authors declare that they have no competing interests.

## Authors' contributions

RGW and BJM conceptualized the manuscript. RGW drafted and developed the manuscript. BJM did extensive reviews of subsequent drafts. RA was involved in editing and formatting the manuscript in various stages. SAB, DS, JDK, NS, DAC, JB, GB and ADW were involved in the early iteration of the manuscript and reviewed and made substantive contributions to the drafts. DS provided crucial data on male circumcision implementation. JBE read and provided insightful comments in the final revisions. All authors have contributed substantively in critically revising the content of the manuscript. All authors have read and approved the manuscript.
